# Supplementary material for: Engineering regulatory networks for complex phenotypes in E. coli
Source: Nat Commun. 2020 Aug 13;11:4050. doi: 10.1038/s41467-020-17721-4 (PMC7426931; doi:10.1038/s41467-020-17721-4)
Supplement: Supplementary file 1 — Supplementary Information [file 41467_2020_17721_MOESM1_ESM.pdf]

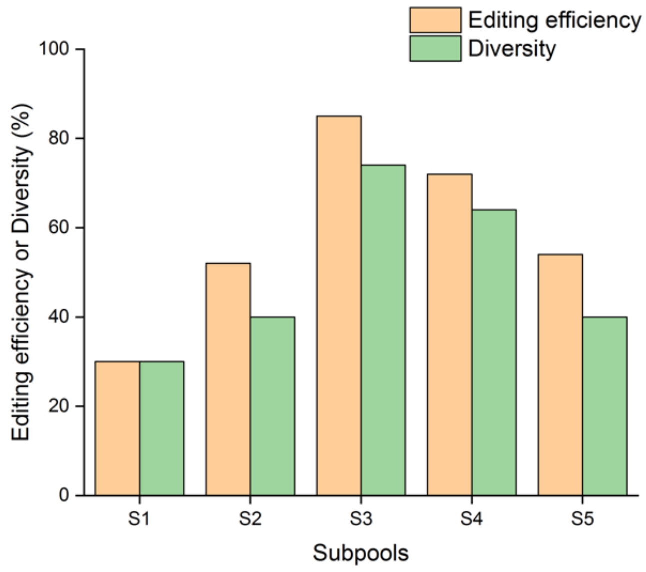

Fig. S1 The editing efficiency and diversity of different Subpools.

The Genome editing efficiency is calculated as follows

$$Genome\ editing\ efficiency = \frac{N}{M} \times 100\%$$

where M denotes the total number of sequenced colonies and N denotes the number of edited colonies (100% matching with the design) found in the sequencing.

The mutational diversity is calculated as follows:

$$Mutational\ diversity = \frac{a}{b} \times 100\%$$

where a denotes the number of different designs found in the sequencing and b denotes the number of total sequenced colonies.

	Homology region	Spacer
Wild-type	GGTAAGTTCAAACTTAACCTCGGTACGCGCGAAATG	
Design	GGTAAGTTCAAACTTGATCTGGGCACCCGCGAAATG	GGAACATTTCGCGCGTACCG
Sequencing data	GGTAAGTTCAAACTTGATCTGGGCACCCGCGAAATG	GGAACATTTCGCGCGTACCG
Wild-type	GGTAAGTTCAAACTTAACCTCGGTACGCGCGAAATG	
Design	GGTAAGTTCAAACTTGATCTGGGCACCCGCGAAATG	GGAACATTTCGCGCGTACCG
Sequencing data	GGTAAGTTCAAACTTAACCTCGGTACGCGCGAAATG	GGAACATTTCGCGCGTAC-G
Wild-type	AGCCATCCGCGTGAGCCGCTCTCCCGCGATAAGCTG	
Design	AGCCATCCGCGTGAGCCGCTCAAAAGAGACAAGCTG	CATGTGTCAATTGCAGAAAA
Sequencing data	AGCCATCCGCGTGAGCCGCTCTCCCGCGATAAGCTG	AAGGTTCATCAGCTTATC--
Wild-type	AGACGAGCCGATGCCGCTCACCAGCGGTGAGTTTGC	
Design	AGACGAGCCGATGCATCTCACGTCTGGCGAGTTTGC	CAGTACCGCAAACTCACCGC
Sequencing data	AGACGAGCCGATGCCGCTCACCAGCGGTGAGTTTGC	CAGTACCGCAAACTCACCG-

Fig. S2 The sequencing data of the colonies harboring the editing plasmid with SNPs.

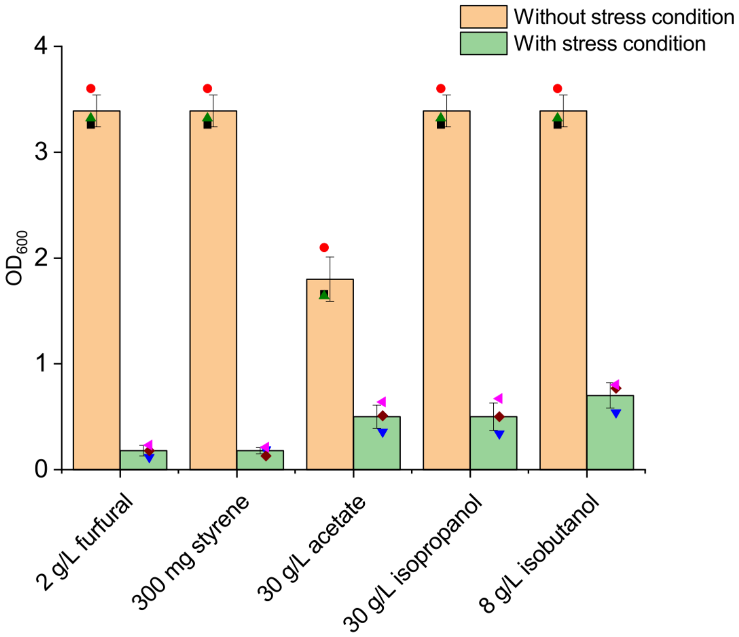

Fig. S3 Growth verification of the non-targeting under different conditions. The acetate selection was performed in the M9 medium, and other selections were performed in the LB medium. $n = 3$ for each curve. Error bars show mean value ± SD.

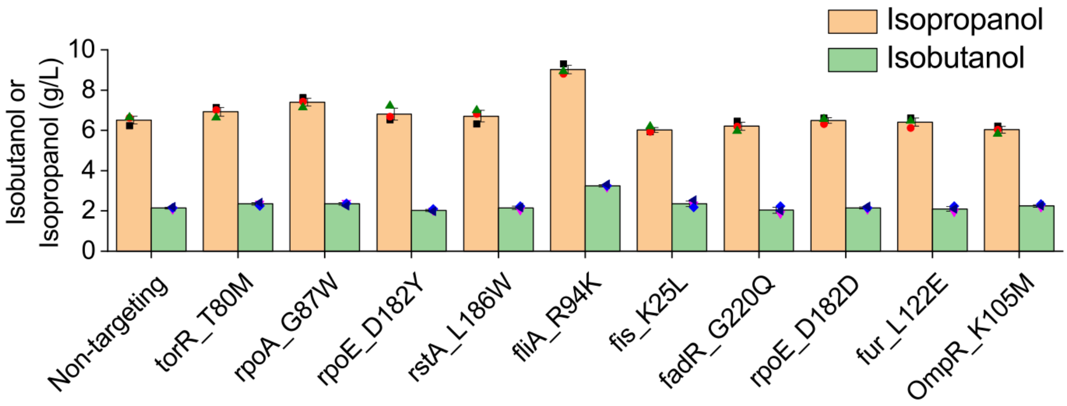

Fig. S4 The isobutanol (48 h) and isopropanol (24 h) flask fermentation using different variants (3 biological replicates). Error bars show mean value ± SD.

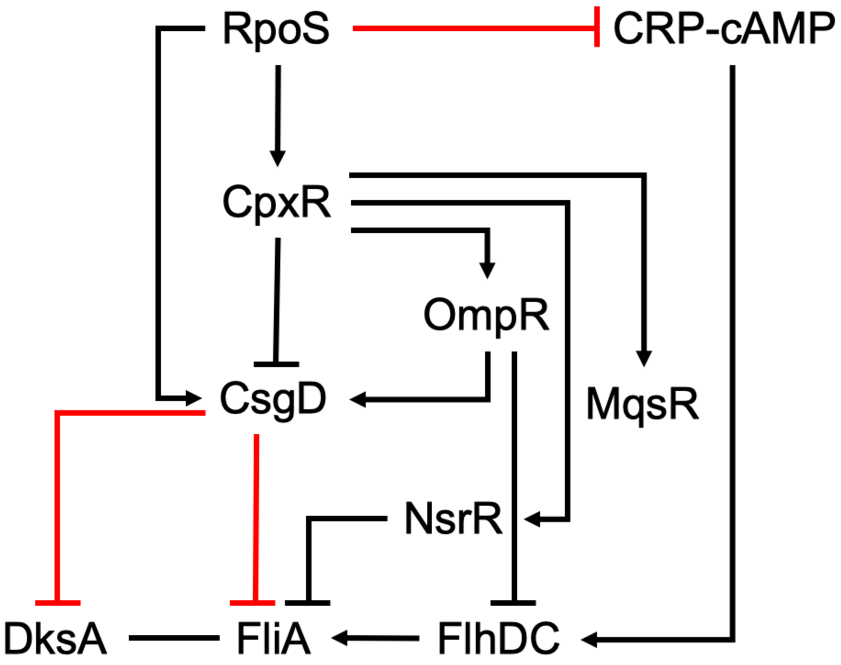

Fig. S5 The regulatory network for FliA. The green line was used for the genes under CpxR regulation. The red line showed the strong repressions.

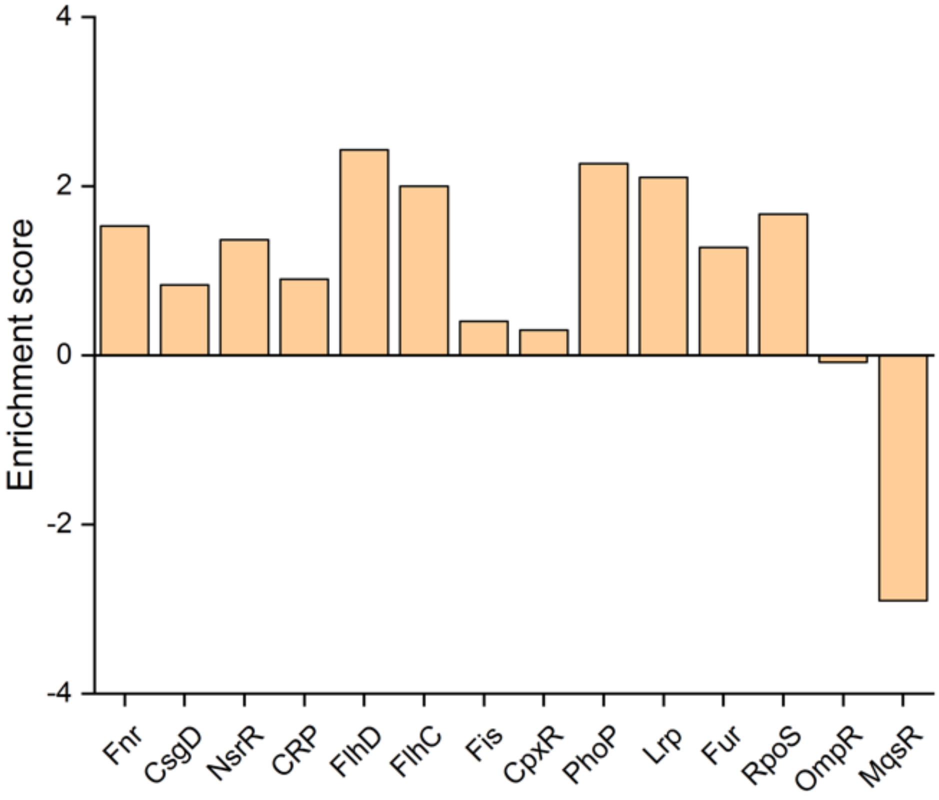

Fig. S6 The enrichment score for the transcriptions of the regulator genes in the mutant FliA_R94K. The transcription change (E_j) was calculated as follows:

$$E_j = \frac{log_2(Y_j)}{log_2(X_j)}$$

where X_j is the transcripts of the WT control and Y_j is the transcripts of the mutants.

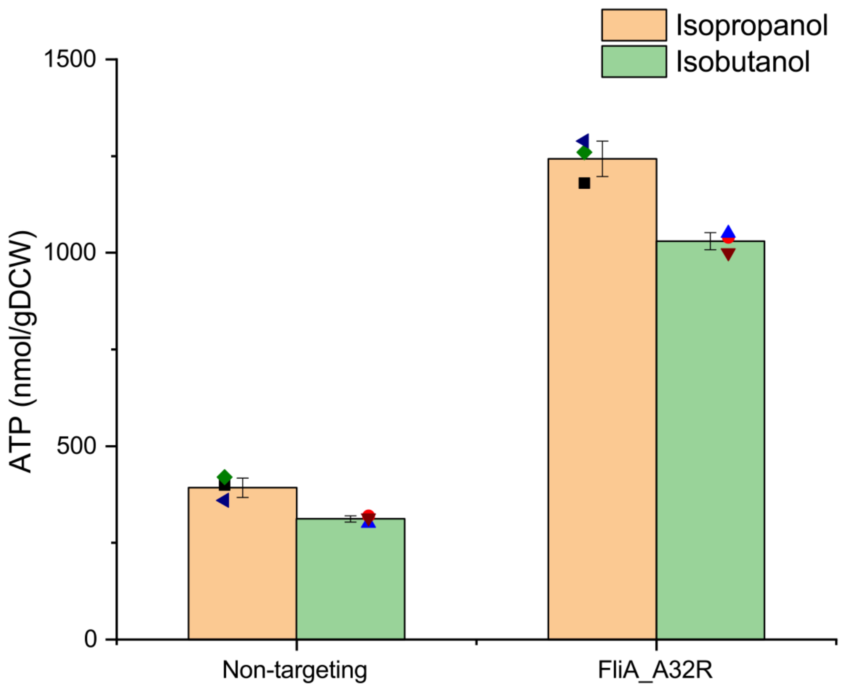

Fig. S7 The ATP concentration test in the mutant FliA_R94K and non-targeting control (3 biological replicates). Error bars show mean value ± SD. The ATP concentration was tested by the BacTiter-GloTM Microbial Cell Viability assay kit.

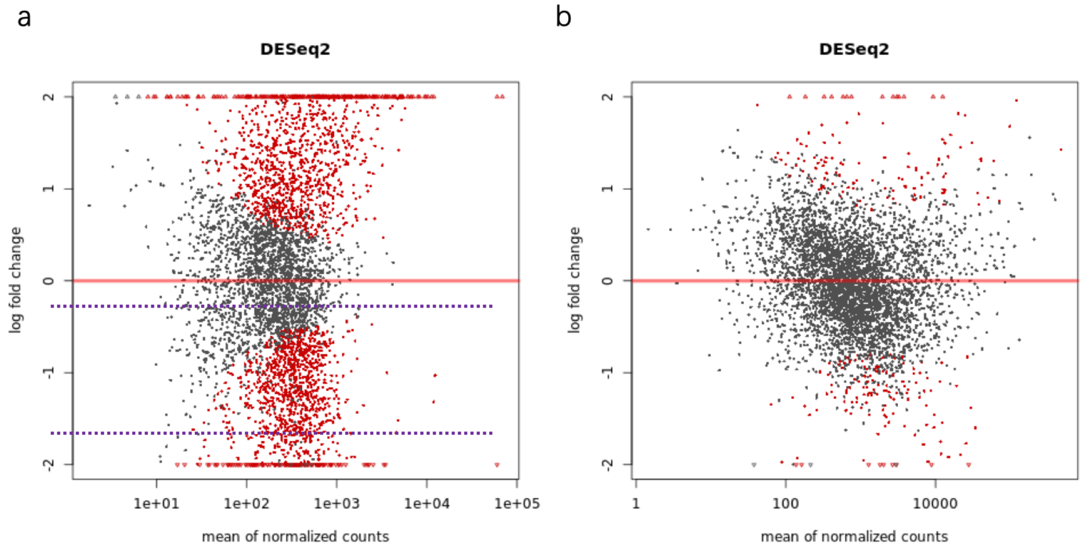

Fig. S8 The DEseq2 plots for the FliA_R94K mutant (a) with or (b) without isobutanol stress. The purple line indicates the cutoff for significantly enriched transcripts (E_j>1 or <-1). The transcription change (E_j) was calculated as follows

$$E_j = \frac{log_2(Y_j)}{log_2(X_j)}$$

where X_j is the transcripts of the WT control and Y_j is the transcripts of the mutants.

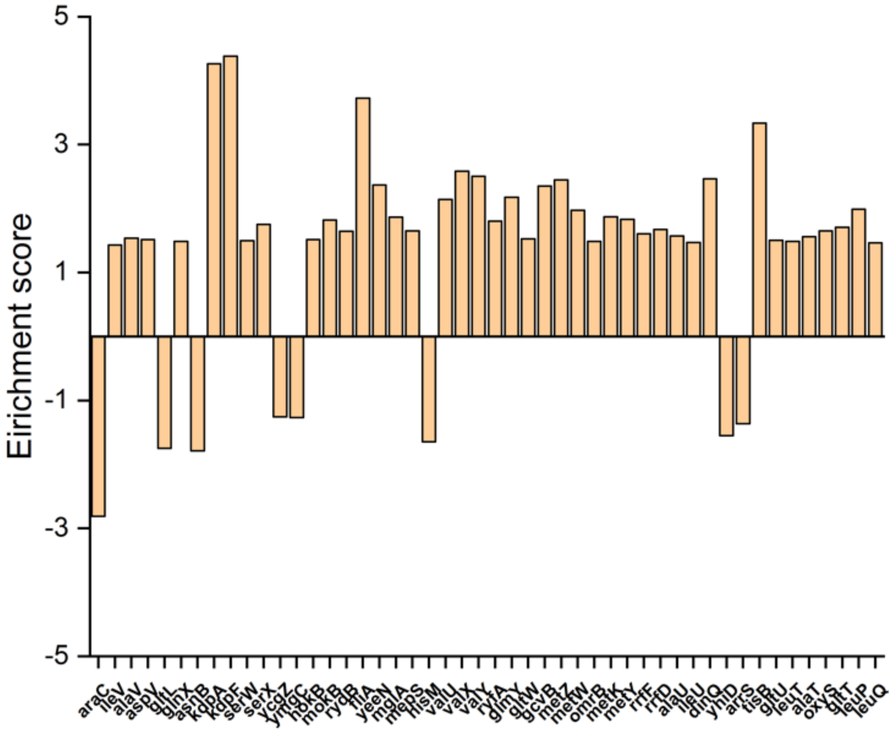

Fig. S9 The enrichment score for the transcriptions of the regulator genes in the mutant FliA_R94K without isobutanol stress. The transcription change (E_j) was calculated as follows:

$$E_j = \frac{log_2(Y_j)}{log_2(X_j)}$$

where X_j is the transcripts of the WT control and Y_j is the transcripts of the mutants.

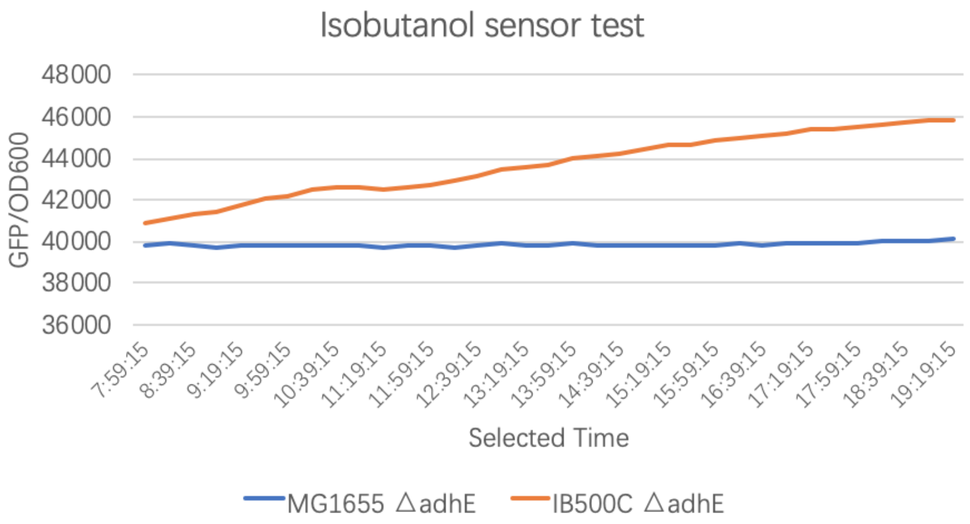

Fig. S10 The pretest of isobutanol sensor in the strain MG1655△adhE and IB500C△ adhE. The data are the average values (3 biological replicates).

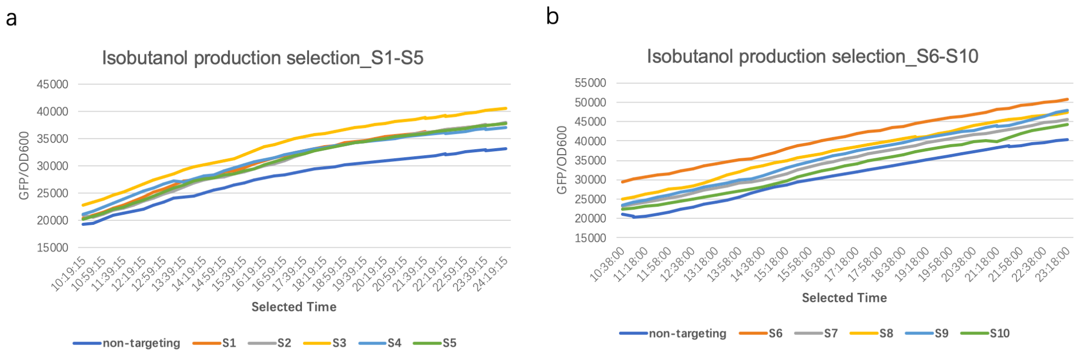

Fig. S11 The assay for IB500C∆adhE with regulator libraries for enhanced isobutanol production. The data are the average values (3 biological replicates).

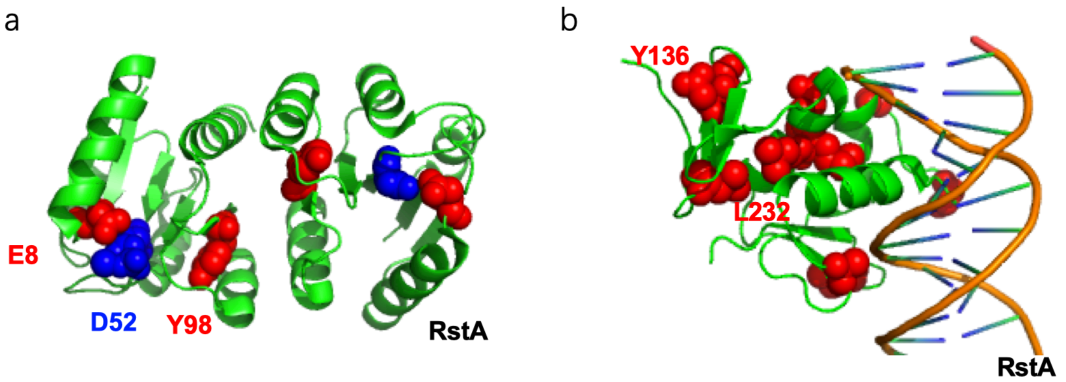

Fig. S12 The predicted structural model of RstA. (a) The predicted structural model of the receiver domain of RstA (PDB: 4NIC). Newly identified mutated residues are shown in red. The D52 residue is shown in blue. **(b)** The predicted structural model of the DNA binding domain in RstA (PDB: 4NHJ). Newly identified mutated residues are shown in red.

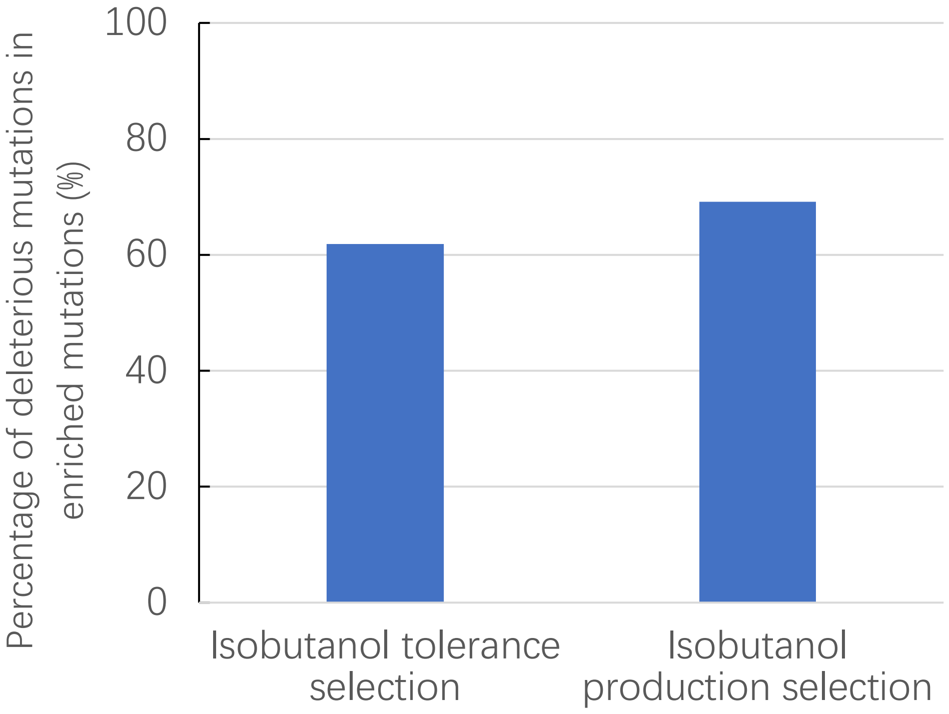

Fig. S13 The evaluation of enriched mutations of *rstA* gene from isobutanol tolerance and production selections using PROVEAN.

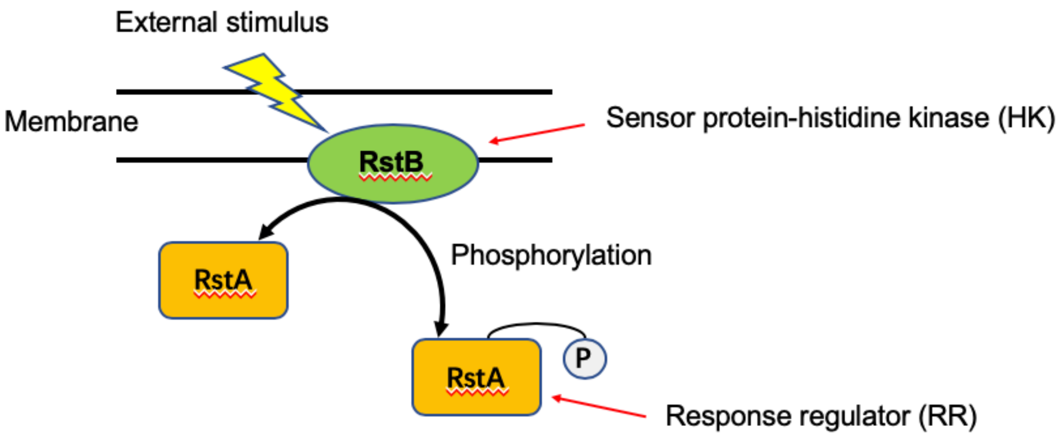

Fig. S14 Schematic depicting the two-component regulatory system (RstA/RstB).

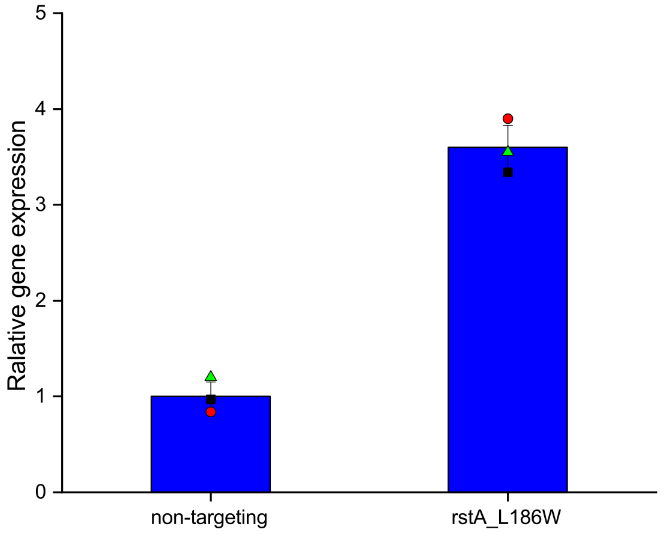

Fig. S15 The relative expression of *csgD* gene in rstA_L186W and non-targeting control (3 biological replicates). Error bars show mean value ± SD.

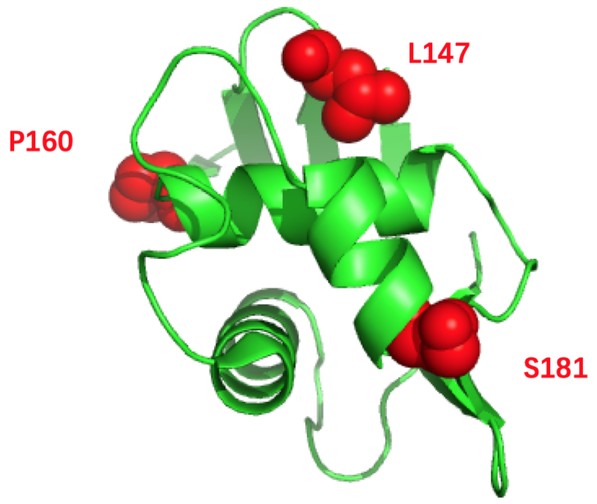

Fig. S16 The structural model of the DNA binding domain of OmpR (PDB: 1OPC). The mutated residue is shown in red.

Table S1 The regulatory network library design

gene	libsize	residues	Subpool
hns	40	12+11	S1
cspA	180	25+26+14+10+36+35+62+64+9	S1
arcA	180	11+10+54+62+103+100+81+104+	S1
fur	260	101+33+88+133+108+89+125+90+93+81+87+96+	S1
narL	240	13+59+14+109+110+67+106+87+164+208+169+165	S1
lrp	160	12+58+17+19+57+56+28+55	S1
mlc	1020	261+258+259+246+264+247+257+153+152+213+335+1	S1
ihfB	40	12+67	S1
cspE	240	11+25+15+14+28+42+35+60+5+6+9+58	S1
crp	1820	176+61+62+63+137+65+113+112+82+83+111+173+87+	S1
phoB	180	10+61+53+102+9+83+106+105+	S1
dnaA	560	235+234+231+177+232+179+178+281+176+175+174+17	S1
rpoS	80	177+174+173+178	S1
rpoE	220	153+152+144+148+133+183+130+126+134+129+188	S1
fnr	840	133+132+131+137+139+138+151+150+154+158+191+4	S1
soxR	620	124+122+130+119+60+114+131+63+111+65+118+44+4	S1
ihfA	140	17+19+50+53+67+68+69	S1
fis	180	25+56+15+23+70+40+63+67+9	S1
cspA	260	11+20+21+22+23+19+18+31+30+60+33+17+32	S1
arcA	2020	216+217+214+215+212+213+210+211+218+219+227+1	S1
narL	900	157+156+159+158+197+196+191+190+193+192+179+17	S1
lrp	660	51+29+60+61+36+53+34+48+49+46+47+44+45+42+43+	S1
mlc	400	91+39+38+98+33+37+42+36+35+41+195+96+40+219+9	S1
ihfB	1600	56+54+42+22+43+60+61+62+63+64+65+66+69+68+83+	S1
cspE	380	24+10+12+59+21+17+23+19+18+31+16+22+29+34+33+	S1
argP	400	24+25+26+27+21+22+23+33+32+31+30+28+39+35+29+	S1
crp	720	179+60+136+135+178+177+139+174+172+171+170+27+	S1
phoB	1980	216+217+214+215+212+213+210+211+218+219+133+1	S2
cytR	580	56+60+62+63+24+25+26+27+20+21+22+23+28+29+40+	S2
dnaA	580	397+399+398+407+405+404+402+401+400+421+420+42	S2
soxS	400	24+25+26+27+43+40+33+32+31+30+28+39+35+41+38+	S2
rpoS	500	310+277+289+288+287+308+300+301+302+303+304+3	S2
rpoN	400	379+372+367+381+380+378+382+383+384+385+368+3	S2
rpoE	720	155+154+157+156+159+158+137+179+178+177+176+17	S2
fnr	440	216+214+215+212+198+210+211+194+197+196+199+21	S2
soxR	620	64+68+69+24+25+26+27+20+21+22+23+46+47+45+28+	S2
ihfA	740	60+61+62+63+64+65+66+82+80+86+87+84+85+24+20+	S2
fis	440	88+89+82+83+80+81+86+87+84+85+77+76+75+74+73+	S2
arcA	80	60+61+57+58	S2
fur	300	121+122+120+132+131+130+111+110+126+127+128+12	S2
narL	80	62+63+65+66	S2
mlc	1160	214+334+336+331+333+332+133+132+137+136+235+1	S2
ihfB	220	39+38+14+17+16+13+35+34+92+94+78	S2
crp	200	155+210+157+156+159+158+209+165+160+161	S2
phoB	80	60+57+56+59	S2
cytR	100	55+54+57+58+61	S2

rpoE	1060	151+150+54+43+132+131+135+181+32+24+25+26+27+	S2
soxR	220	59+66+67+99+76+62+109+113+112+95+105	S2
ihfA	920	43+89+83+81+25+26+27+21+22+44+42+29+40+41+3+7	S2
fis	840	42+50+60+61+62+64+53+66+69+52+24+26+27+48+49+	S2
hns	400	133+65+66+109+127+107+120+114+117+130+116+110+	S3
cspA	420	42+29+61+63+27+48+46+47+43+41+7+6+8+15+13+12+	S3
arcA	120	55+56+117+113+82+105	S3
fur	340	59+58+17+55+19+54+57+56+51+53+52+60+61+62+63+	S3
narL	1340	199+154+211+115+132+131+130+64+53+83+82+212+8	S3
lrp	680	66+134+133+132+62+63+64+136+135+67+68+139+138	S3
mlc	1280	319+215+198+211+158+317+316+131+318+135+178+27	S3
cspE	260	39+38+55+57+56+13+40+43+26+53+7+8+44	S3
argP	520	229+218+219+114+63+111+110+230+118+232+233+107	S3
crp	100	76+144+28+125+23	S3
phoB	120	55+54+115+91+107+84	S3
cytR	440	153+154+196+102+69+82+253+81+198+44+79+281+20	S3
dnaA	40	269+180	S3
soxS	80	52+49+51+23	S3
rpoS	160	143+99+62+229+227+101+231+232	S3
fnr	360	91+59+169+55+78+99+102+117+109+111+110+228+22	S3
soxR	420	51+36+61+88+35+87+49+40+38+73+91+90+93+79+39+	S3
ihfA	60	13+59+70	S3
fis	120	20+22+46+19+68+8	S3
rpoH	80	55+82+88+20	S3
nsrR	60	102+91+96	S4
basR	180	59+51+98+7+102+8+101+79+	S4
rutR	220	77+101+115+74+97+179+134+176+167+171+78	S4
ompR	160	11+12+55+63+102+83+106+105	S4
rstA	140	52+60+98+9+8+101+79	S4
rcsB	160	11+10+56+109+110+67+106+87	S4
dksA	120	114+117+138+135+74+71	S4
phoP	160	59+51+98+7+102+8+101+79	S4
oxyR	40	208+199	S4
evgA	160	99+52+60+7+103+102+8+80	S4
torR	160	10+53+99+61+9+103+80+102	S4
fadR	320	216+217+213+106+105+219+99+107+179+102+103+10	S4
fliA	220	81+98+96+74+73+91+92+95+94+84+78	S5
cpxR	160	59+9+8+100+101+97+78+51	S5
narP	160	13+59+14+109+110+67+106+87	S5
ntrC	560	234+62+238+239+82+235+236+174+173+172+171+170+	S5
flhD	160	82+2+91+83+92+94+84+96	S5
flhC	180	144+140+157+141+156+137+91+139+160	S5
lexA	40	119+156	S5
modE	1200	126+127+161+182+183+162+184+185+128+166+256+2	S5
fhlA	380	472+474+480+473+481+475+414+415+416+541+410+47	S5
argR	120	21+47+44+76+123+105	S5
iscR	60	98+92+104	S5
purR	120	71+196+124+190+74+147	S5

metJ	340	39+71+3+44+70+43+60+61+62+63+64+65+66+67+68+2	S5
rcsA	20	145	S5
hyfR	380	380+381+443+442+441+440+447+446+445+444+379+37	S5
tyrR	640	154+115+131+130+137+239+234+235+236+237+238+16	S5
dpiA	160	11+12+57+108+65+107+104+85	S5
malT	160	39+46+44+45+42+43+40+41	S5
nsrR	580	28+43+60+61+26+48+49+46+47+44+45+42+29+40+41+	S6
cra	580	30+20+21+22+47+3+5+4+7+6+9+8+11+10+13+12+15+1	S6
basR	1900	216+217+214+215+212+213+210+211+218+133+132+13	S6
rutR	400	52+39+45+58+48+49+46+47+44+56+51+43+40+42+55+	S6
ompR	2000	216+217+214+215+212+213+210+211+218+219+227+13	S6
rstA	2000	216+217+214+215+212+213+210+211+218+219+227+13	S6
gadE	400	146+147+144+145+142+143+140+141+150+151+148+14	S6
gadX	400	167+162+165+173+163+161+164+180+168+169+179+17	S6
rcsB	500	153+152+198+179+178+177+176+175+174+173+172+17	S6
gadW	400	167+155+157+156+159+158+160+161+163+168+169+16	S6
phoP	1980	216+217+214+215+212+213+210+211+218+219+133+13	S6
oxyR	400	24+25+26+27+20+21+22+23+19+18+31+30+28+29+35+	S6
evgA	500	191+179+178+177+176+175+174+173+172+171+170+18	S6
torR	1920	216+217+214+215+212+213+210+211+218+219+133+13	S6
fadR	680	37+36+63+64+35+66+67+68+69+34+48+49+46+47+44+	S6
marA	1160	30+36+42+43+88+89+35+90+82+83+80+81+86+87+84+	S6
fecI	500	151+150+153+152+155+154+157+156+159+158+132+13	S6
fliA	500	216+217+214+215+212+213+210+211+190+218+219+20	S7
cpxR	2000	216+217+214+215+212+213+210+211+218+219+133+13	S7
narP	500	155+157+156+190+179+178+177+176+175+174+173+17	S7
ntrC	400	458+459+449+448+457+450+451+452+453+454+446+44	S7
lexA	1000	36+28+29+60+61+62+63+64+35+66+34+24+25+26+27+	S7
modE	940	51+36+60+61+62+63+64+35+66+67+68+69+34+48+49+	S7
cysB	400	24+25+26+27+20+21+22+23+19+32+31+30+28+29+35+	S7
csgD	500	157+159+158+191+190+192+179+178+177+176+175+17	S7
pdhR	580	37+35+66+67+69+48+49+46+47+44+45+42+43+40+41+	S7
nagC	200	39+38+42+44+37+43+35+36+40+41	S7
fhlA	400	664+678+679+669+668+667+666+665+677+670+671+66	S7
iscR	1080	30+28+43+60+61+62+63+64+65+66+83+86+87+84+85+	S7
purR	860	54+192+115+275+26+27+20+21+22+23+47+44+45+28+	S7
rob	600	42+43+83+34+87+24+25+26+27+21+49+28+29+40+41+	S7
metJ	700	56+42+53+52+24+25+26+27+20+21+22+23+45+28+29+	S7
nac	400	24+25+26+27+20+21+22+23+19+18+31+30+28+29+35+	S7
leuO	400	52+39+45+58+48+49+46+47+44+56+51+43+40+42+55+	S7
rcsA	500	155+154+157+156+159+158+139+174+173+172+171+17	S7
hyfR	400	641+647+653+646+643+660+656+657+654+642+652+64	S7
tyrR	400	493+484+492+502+483+491+487+486+498+499+489+48	S7
dpiA	400	199+198+195+194+197+196+191+190+193+192+182+18	S7
nanR	580	60+61+62+63+64+65+66+67+68+69+87+77+76+75+74+	S7
galR	580	54+20+21+22+23+45+28+29+5+4+7+6+9+8+14+11+10+	S7
galS	580	54+20+21+22+23+45+28+29+5+4+7+6+9+8+14+11+10+	S7
agaR	400	39+38+41+48+49+33+32+44+42+51+43+35+36+46+37+	S7

gntR	500	24+25+26+27+20+21+22+23+9+8+11+10+13+12+15+14	S7
trpR	1020	68+88+89+64+65+66+67+82+83+80+81+86+87+84+85+	S7
araC	400	201+200+203+202+199+198+207+206+209+208+197+21	S7
yiaJ	400	45+59+58+48+49+46+47+57+56+51+50+53+52+60+55+	S7
malT	500	883+858+854+855+856+857+837+852+853+838+839+87	S7
appY	400	167+155+157+156+159+158+160+161+163+168+169+16	S7
cra	100	55+52+51+48+49	S7
basR	80	55+54+57+58	S7
ompR	80	61+62+59+58	S7
rstA	80	55+56+59+58	S7
phoP	80	55+54+57+58	S7
evgA	80	55+56+59+58	S7
torR	80	60+57+56+59	S7
marA	320	13+12+15+14+17+16+19+18+23+121+0+3+4+9+20+21	S7
cpxR	80	55+54+57+58	S8
narP	80	62+63+65+66	S8
ntrC	80	60+61+57+58	S8
flhD	320	77+38+59+33+31+30+28+29+35+34+60+61+62+74+40+	S8
flhC	660	159+158+134+68+88+89+97+67+82+83+81+86+87+85+	S8
purR	20	46	S8
dpiA	80	60+61+63+64	S8
galR	440	61+110+68+254+49+46+47+82+222+79+281+280+74+9	S8
galS	440	61+110+68+254+49+46+47+82+222+79+281+280+74+9	S8
gntR	80	54+51+50+53	S8
nsrR	120	59+2+5+4+6+9	S8
cra	820	210+156+159+158+197+88+116+274+82+81+119+118+8	S8
basR	120	23+53+116+103+80+106	S8
ompR	120	57+56+110+101+107+84	S8
rstA	160	11+12+57+62+70+82+83+104	S8
fliZ	340	84+23+37+51+40+61+108+109+168+65+165+68+129+1	S8
gadE	180	48+46+56+74+73+7+6+8+90	S8
gadX	160	11+200+203+210+234+242+9+232	S8
rcsB	260	200+203+199+58+23+117+88+111+102+165+105+86+1	S8
gadW	120	197+5+7+228+226+236	S8
dksA	260	39+48+49+47+44+42+51+43+99+98+100+107+96	S8
phoP	60	103+80+53	S8
oxyR	600	126+219+133+114+137+110+103+230+232+233+106+4	S8
evgA	340	200+153+101+199+156+158+196+193+192+98+187+66+	S8
torR	180	55+54+117+88+104+83+81+84+85	S8
fecI	40	123+124	S8
cpxR	120	10+32+53+52+66+102	S9
narP	320	203+202+210+206+209+61+88+111+70+68+168+103+1	S9
ntrC	280	142+140+141+51+357+321+325+281+280+358+365+10	S9
cysB	880	150+229+228+133+102+63+112+236+100+232+233+224	S9
csgD	220	59+204+211+208+61+168+110+18+212+205+170	S9
pdhR	20	11	S9
nagC	260	155+157+156+195+194+196+244+247+266+342+225+22	S9
fdhF	40	417+522	S9

argR	200	27+113+131+114+108+109+66+90+106+87	S9
purR	700	195+191+114+89+274+113+279+278+69+251+86+255+9	S9
metJ	140	11+10+12+33+32+36+9	S9
nac	500	216+217+127+218+219+115+111+135+112+119+231+23	S9
leuO	720	210+132+131+238+239+135+134+252+139+237+119+13	S9
rcsA	300	150+152+194+57+190+193+186+187+7+54+9+8+106+84	S9
hyfR	40	383+488	S9
tyrR	260	10+207+206+208+190+380+416+417+384+242+9+420+3	S9
dpiA	80	68+86+109+59	S9
nanR	80	32+57+89+50	S9
galR	260	146+145+194+190+219+245+122+273+73+71+67+188+2	S9
galS	260	146+145+194+190+219+245+122+273+73+71+67+188+2	S9
gntR	720	150+126+197+114+117+259+111+275+252+83+80+81+8	S9
yiaJ	200	152+212+157+159+158+193+241+239+222+220	S9
malT	140	891+892+850+885+884+888+848	S9
appY	120	197+5+7+228+226+236	S9
marR	1020	11+12+13+14+15+16+17+18+19+20+21+22+23+24+25+	S10
rpoA	1140	216+198+210+313+197+196+190+192+133+273+275+13	S10
rpoC	4880	339+334+311+795+794+791+790+939+346+938+933+93	S10
rpoD	840	594+596+452+551+553+390+395+494+497+490+183+54	S10
rho	1480	158+179+178+174+173+172+171+170+182+183+180+18	S10
rpoH	20	80	S10
rpoA	300	295+265+300+264+293+263+298+299+296+297+269+26	S10
rpoB	560	1269+1268+1267+60+62+390+179+177+473+183+180+2	S10
rpoC	640	1269+1268+1326+1327+1148+1267+60+62+390+179+17	S10
rpoD	1120	591+590+593+578+595+573+572+571+453+577+576+57	S10
rho	580	212+62+64+110+66+112+83+80+288+326+283+78+285+	S10
rpoH	500	265+270+271+258+259+275+252+253+256+257+254+23	S10
rpoB	2500	1304+1305+768+769+1301+1302+1263+1308+1306+126	S10
rpoB	2760	927+1227+1226+1225+1224+1223+1222+1221+1220+13	S10
rpoD	1440	605+604+601+600+597+602+450+451+608+518+499+49	S10
rho	320	308+339+338+334+302+336+305+333+381+340+385+34	S10

Table S2 The strains, plasmids, oligos, and primers used in this study

Name	Sequence	Notes
PA14		23
IB500C		24
pSIM5		https://redrecombineering.ncifcrf.gov/
pX2-Cas9	https://benchling.com/s/3c941j	
Editing plasmid for galKoff	https://benchling.com/s/seq-NUfPfoa8VRgzg9tiebMG	
rpoB_E546C	CCTGTCTTGGCGGACACACACAAACGTCGTATCTCCGCACTCGGCCCAGGCGGTCTGACGCGTGAACGTGCAGGCTTCTGCGTTCGAGACGTACACCCGACTCACTACGGTCGCGTAGATCTTGACAGCTAGCTCAGTCCTAGGTATAATACTAGTTTCGAAGCCTGCACGTTCACGTTTTAGAGCTAGAAATAGCAAGT	
soxR_I120A	CCCTGAGTCCTCCGCTGCGGCGAGAAGAGTTGGATCGGCGCATTCATACCTTAGTTGCGCTGCGTGACGAACTGGACGGATGTGCGGGTTGTGGCTGCCTTTCGCGCAGTGATTGCCGATCTTGACAGCTAGCTCAGTCCTAGGTATAATACTAGTTCGGCGCATTCATACCTTAGGTTTTAGAGCTAGAAATAGCAAGT	Editing cassette for furfural experiments
rpoC Q623R		
marR_K81T	AAGAGCACCGCCTTCGTCTGAACACCTGCTACGTTCGGAAGGTGCCCTCAGTCGACCTGGGAGCACTGACCCGTATGCTGGATCGCCTGGTCTGTACCGGCTGGGTTGAAAGGTTGCCGAACCCGAATGACAAGCGCGGCGTACTGGTAGATCTTGACAGCTAGCTCAGTCCTAGGTATAATACTAGTCCTGGTCTGTAAAGGCTGGGGTTTTAGAGCTAGAAATAGCAAGT	
csgD_L180F	GCGACGCACCTTCCCATGCAGTGAGTATCCTCCTGAATAAGCTGCGTATCGGCGCGTCTAATAACGAGATCGCTCGTTCGTTTTTCATCAGCGAGAACACCGTTAAAACGCATCTTTATAATCTTTTCAAGAAGATAGCCGTAAAAAATTGACAGCTAGCTCAGTCCTAGGTATAATACTAGTATCGTTGTTCATCAGCGAAAATAGTTTTAGAGCTAGAAATAGCAAGT	

fliA_E215V	GCGACGCACCTTCCCATGAGAGATCGAGAGTGGTATTAACCCTCTATTACCAGGAAGAGCTGAATCTCAAAGAGATTGGCGCGGTTTTAGTGGTCGGGGAATCGCGGGTCAGTCAGTTACACAGCCAGGCTATTAAACGGTTACGCACTTGACAGCTAGCTCAGTCCTAGGTATAATACTAGTATCAGAGATTGGCGCGGTGCTGGGTTTTAGAGCTAGAAATAGCAAGT	Editing cassette for styrene experiments
lexA_L35M	GCGACGCACCTTCCCATGGACTAGAGTAGCTCCGTGATCACATCAGCCAGACAGGTATGCCGCCGACGCGTGCGGAAATCGCGCAAAGAATGGGGTTCCGTTCCCCAAACGCGGCTGAAGAACATCTGAAGGCGCTGGCACGCAAAGGTTGACAGCTAGCTCAGTCCTAGGTATAATACTAGTATCTGCGGAAATCGCGCAGCGTTGTTTTAGAGCTAGAAATAGCAAGT	
narP_S170E	GCGACGCACCTTCCCATGCTCGTGACCAGAATCCCTTCAGCGTGCTGACGGAGCGCGAGCTGGATGTTCTGCACGAGTTAGCGCAAGGGCTGGAAAATAAACAGATTGCCTCGGTGTTGAATATTTCCGAGCAGACAGTAAAAGTACATTGACAGCTAGCTCAGTCCTAGGTATAATACTAGTATCTGTTCTGCACGAGCTGGCACGTTTTAGAGCTAGAAATAGCAAGT	
soxR_V23Y	CCTGTCTTGGCGGACACATCGGCAGATCTGAGAAATTACCCCGCATTAAAGCGCTGCTAACCCCCGGCGAAGTGGCGAAACGCTCTGGCTATGCGGTATCGGCGCTGCATTTCTATGAAAGTAAAGGGTTGATTACCAGTATCCGTAATTGACAGCTAGCTCAGTCCTAGGTATAATACTAGTATCAGTGGCGAAACGCAGCGGTGGTTTTAGAGCTAGAAATAGCAAGT	
rpoS_E289T	CCTGTCTTGGCGGACACATGAGTCGAGGTAAACAGCGTGAAGTGCTGGCACGTCGATTCGGTTTGCTGGGGTACGAAGCGGCGACCTTAACCGATGTAGGTCGTGAAATTGGCCTCACCCGTGAACGTGTTCGCCAGATTCAGGTTGATTGACAGCTAGCTCAGTCCTAGGTATAATACTAGTATCGGGGTACGAAGCGGCAACACGTTTTAGAGCTAGAAATAGCAAGT	Editing cassette for acetate

		for acetate experiments
cytR_L17Q	CCTGTCTTGGCGGACACAAGTTGATGACGAGAGGAGTGAGTGTGAAAGCGAAGAAGCAGGAAACTGCCGCGACCATGAAAGACGTTGCGCAGAAAGCAAAAGTCTCTACAGCGACCGTCTCCCGAGCATTAATGAATCCCGATAAAGTTTGACAGCTAGCTCAGTCCTAGGTATAATACTAGTATCTGTAGAGACTTTTGCCTTGAGTTTTAGAGCTAGAAATAGCAAGT	
phoB_R213G	CCTGTCTTGGCGGACACAATCGCTCACAAGGCACGGTCGATGTCCACATTCGTCGCCTGCGTAAAGCACTGGAGCCCGGCGGGCATGACGGCATGGTTCAGACCGTGCGCGGTACAGGATATCGTTTTTCAACCCGCTTTTAACGCCTTTGACAGCTAGCTCAGTCCTAGGTATAATACTAGTATCCGCGCACGGTCTGCACCATGGTTTTAGAGCTAGAAATAGCAAGTT	
torR_T80M	GCGGTGGCATTTTCAGGGACGAAGTGATCGATGGCCTGATGTTAACCCGCGCCCTGCGAGAACGCTCAACGGTGGGGATTATTCTGGTTATGGGCAGAAGCGATCGGATTGACCGTATTGTTGGGCTGGAAATGGGCGCAGACGATTATTGACAGCTAGCTCAGTCCTAGGTATAATACTAGTATCGTCAATCCGATCGCTGCGTCGTTTTAGAGCTAGAAATAGCAAGT	
rpoA_G87W	GGCGAAAGCGGCTGAAATCTGCAGGTACTGAGTACAGCACCAAAGAAGGCGTTCAGGAAGATATCCTGGAAATCCTGCTCAACTTAAAGTGGCTGGCGGTGAGAGTTCAGGGCAAAGATGAAGTTATTCTTACCTTGAATAAATCTGGTTGACAGCTAGCTCAGTCCTAGGTATAATACTAGTATCGAAATCCTGCTCAACCTGAAGTTTTAGAGCTAGAAATAGCAAGT	
rpoE_D182Y	AAGAGCACCGCCTTCGTCTCGACGTATCGACGCTATCATGGATTGTCCGGTAGGTACGGTGCGTTCACGTATCTTCAGGGCCCGTGAAGCTATTTATAACAAAGTTCAACCGCTTATCAGGCGTTGACGATAGCGGGATACTGGATAATTGACAGCTAGCTCAGTCCTAGGTATAATACTAGTATCGTTCACGTATCTTCCGAGCGGTTTTAGAGCTAGAAATAGCAAGT	

rstA_L186W	GAATCCTCCCGAGCCCACTGCGCTGTGTGAGCTGATTTCGAATTATTGTGGGAATTAGCTACCCATGCCGGGCAAATCATGGACCGTGACGCGTGGCTGAAAAATTTACGCGGCGTCAGTTATGACGGACTGGATCGTAGCGTGGACGTTGACAGCTAGCTCAGTCCTAGGTATAATACTAGTATCAATTTTTCAGCAATGCATCGGTTTTAGAGCTAGAAATAGCAAGT	Editing cassette for isopropanol and isobutanol tolerance selection experiments
fliA_R94K	AGCCGGCGGAGTCTCCGTACTTCTGTCACAGCAGCGTATCCGTGGCGCTATGCTGGATGAACTTCGCAGCCGTGACTGGGTGCCAAGATCTGTGAAACGCAACGCGCGTGAAGTGGCACAGGCAATAGGGCAACTGGAGCAGGAACTTTTGACAGCTAGCTCAGTCCTAGGTATAATACTAGTATCGCGTTGCGTCGCACGCTGCGGTTTTAGAGCTAGAAATAGCAAGT	
fis_K25L	TCCTCTGGCGGAAAGCCTGCGTGCAAGATGCGTAAATTCTGACGTACTGACCGTTTCTACCGTTAACTCTCAGGATCAGGTAACCCAACTGCCGTTAAGAGACTCGGTTAAACAGGCACTGAAGAACTATTTTGCTCAACTGAATGGTTTGACAGCTAGCTCAGTCCTAGGTATAATACTAGTATCTGTTTAACCGAGTCACGCAGGTTTTAGAGCTAGAAATAGCAAGT	
fadR_G220Q	GCGGTGGCATTTTCAGGGTATCGACATACGGCAGTGAAGGCGCGCACGATCAGGTGTACGAAACAGTGCGTCGCTATGGGCATGAATCACAGGAGATTTGGCACCGGATGCAGAAAAATCTGCCGGGTGATTTAGCCATTCAGGGGCGTTGACAGCTAGCTCAGTCCTAGGTATAATACTAGTATCCGTCGCTATGGGCATGAGAGGTTTTAGAGCTAGAAATAGCAAGT	
rpoE_D182D	AAGAGCACCGCCTTCGTCACGAGCTACACTCGCTATCATGGATTGTCCGGTAGGTACGGTGCGTTCACGTATCTTCAGGGCCCGTGAAGCTATTGATAACAAAGTTCAACCGCTTATCAGGCGTTGACGATAGCGGGATACTGGATAATTGACAGCTAGCTCAGTCCTAGGTATAATACTAGTATCGTTCACGTATCTTCCGAGCGGTTTTAGAGCTAGAAATAGCAAGT	

fur_L122E	AAGAGCACCGCCTTCGTCTCGCTATAGGTATT AGTGATGATTCCATCGAAGCGCGTCAGCGTG AAATTGCCGCAAAACATGGCATTCGCGAAAC CAATCACAGTCTCTATCTTTACGGTCACTGTGC CGAAGGCGATTGCCGCGAAGATTGACAGCTA GCTCAGTCCTAGGTATAATACTAGTATCGATA GAGACTGTGGTTAGTCGTTTTAGAGCTAGAAA TAGCAAGT
ompR_K105M	GCGGTGGCATTTTCAGGGCAGCGTCACACAA AAGGGGAAGAAGTGGACCGTATCGTAGGCCT GGAGATTGGCGCTGACGACTACATTCCGATGC CATTTAACCCGCGTGAACTGCTGGCCCGTATC CGTGCGGTGCTGCGTCGTCAGGTTGACAGCTA GCTCAGTCCTAGGTATAATACTAGTATCTCAC GCGGGTTAAACGGTTTGTTTTAGAGCTAGAAA TAGCAAGT
soxR_I120R	TCCTCTGGCGGAAAGCCTGCAATCAGTCTGAA GAGTTGGATCGGCGCATTCATACCTTAGTGGC GCTGCGTGACGAACTGGACGGATGCCGTGGC TGTGGCTGCCTTTCGCGCAGTGATTGCCCGTTG CGTAACCCGGGCGACCGCTTTTGACAGCTAGC TCAGTCCTAGGTATAATACTAGTATCGACGAA CTGGACGGATGTATGTTTTAGAGCTAGAAATA GCAAGT
soxR_S126Q	TCCTCTGGCGGAAAGCCTAGCTCGTAACTGAT TCATACCTTAGTGGCGCTGCGTGACGAACTGG ACGGATGTATTGGTTGTGGCTGCCTGCAGAGA AGTGATTGCCCGTTGCGTAACCCGGGCGACCG CTTAGGAGAAGAAGGTACCGTTGACAGCTAG CTCAGTCCTAGGTATAATACTAGTATCACGGG CAATCACTGCGCGAAGTTTTAGAGCTAGAAAT AGCAAGT
mlc_T16V	AAGAGCACCGCCTTCGTCAGTATGTCACCAAT AGGGAGTATGCGGTGGTTGCTGAAAACCAGC CTGGGCACATTGATCAAATAAAGCAGGTGAA TGCCGGCGCGGTTTATCGCCTGATTGATCAGC TTGGTCCAGTCTCGCGTATCGATTGACAGCTA GCTCAGTCCTAGGTATAATACTAGTATCGCGA TAAACCGCGCCCGCGTGTTTTAGAGCTAGAAA TAGCAAGT

mlc_T16P	AAGAGCACCGCCTTCGTCACTACTCAAGACATAGGGAGTATGCGGTGGTTGCTGAAAACCAGCCTGGGCACATTGATCAAATAAAGCAGCCGAATGCCGGCGCGGTTTATCGCCTGATTGATCAGCTTGGTCCAGTCTCGCGTATCGATTGACAGCTAGCTCAGTCCTAGGTATAATACTAGTATCGCGATAAACCGCGCCCGCGTGTTTTAGAGCTAGAAATAGCAAGT	Editing cassette for isobutanol production selection experiments
ntrC_F237D	AGCCGGCGGAGTCTCCGTAGATTCGTCTAGTTACTGGCGCGAATACCATTCGTCAGGGGCGTTTTGAACAGGCCGATGGCGGTACATTAGATCTGGATGAGATTGGTGATATGCCGCTGGATGTGCAGACGCGTTTGCTGCGCGTGCTTTGACAGCTAGCTCAGTCCTAGGTATAATACTAGTATCGCATATCACCAATTTCGTCGGTTTTAGAGCTAGAAATAGCAAGT	
ntrC_F237L	AGCCGGCGGAGTCTCCGTATGTTACGACACTTACTGGCGCGAATACCATTCGTCAGGGGCGTTTTGAACAGGCCGATGGCGGTACATTACTGCTGGATGAGATTGGTGATATGCCGCTGGATGTGCAGACGCGTTTGCTGCGCGTGCTTTGACAGCTAGCTCAGTCCTAGGTATAATACTAGTATCGCATATCACCAATTTCGTCGGTTTTAGAGCTAGAAATAGCAAGT	
cpxR_L181V	GCGACGCACCTTCCCATGTGCATGACGCATTTTACCCTGCTCTATTTGCTGGCACAGCATCTGGGTCAGGTGGTTTCCCGTGAACATGTGAGCCAAGAGGTTTTGGGCAAACGCCTGACGCCTTTCGACCGCGCTATTGATATGCACATTGACAGCTAGCTCAGTCCTAGGTATAATACTAGTATCGGCGTTTGCCCAACACTTCCGTTTTAGAGCTAGAAATAGCAAGT	
ompR_P160H	GAATCCTCCCGAGCCCACAGCTCTTCATGCGTAAGTTCAAACTTAACCTCGGTACGCGCGAAATGTTCCGCGAAGACGAGCCGATGCATCTCACGTCTGGCGAGTTTGCGGTACTGAAGGCACTGGTCAGCCATCCGCGTGAGCCGCTTTGACAGCTAGCTCAGTCCTAGGTATAATACTAGTATCCAGTACCGCAAACTCACCGCGTTTTAGAGCTAGAAATAGCAAGT	

ompR_P160T	GAATCCTCCCGAGCCCACTGCTTGAGTACGGTAAGTTCAAACTTAACCTCGGTACGCGCGAAATGTTCCGCGAAGACGAGCCGATGACCCTCACGTCTGGCGAGTTTGCGGTACTGAAGGCACTGGTCAGCCATCCGCGTGAGCCGCTTTGACAGCTAGCTCAGTCCTAGGTATAATACTAGTATCCAGTACCGCAAACTCACCGCGTTTTAGAGCTAGAAATAGCAAGT	
ompR_L147D	GAATCCTCCCGAGCCCACGTCGCACAGAAGTGCCAGGCGCACCGTCACAGGAAGAGGCGGTAATTGCTTTCGGTAAGTTCAAACTTAACGATGGCACCCGCGAAATGTTCCGCGAAGACGAGCCGATGCCGCTCACCAGCGGTGAGTTTTGACAGCTAGCTCAGTCCTAGGTATAATACTAGTATCGGAACATTTCGCGCGTACCGGTTTTAGAGCTAGAAATAGCAAGT	
ompR_S181K	GAATCCTCCCGAGCCCACCATGTACGCATGTCACCAGCGGTGAGTTTGCGGTACTGAAGGCACTGGTCAGCCATCCGCGTGAGCCGCTCAAAAGAGACAAGCTGATGAACCTTGCCCGTGGTCGTGAATATTCCGCAATGGAACGCTCTTGACAGCTAGCTCAGTCCTAGGTATAATACTAGTATCAAGGTTCATCAGCTTATCGCGTTTTAGAGCTAGAAATAGCAAGT	
rstA_N150L	GAATCCTCCCGAGCCCACCTGAGCTAAGTCAAACGTCTCTGACTCCCTACAAAGCCCTGCATTTCGGCACGTTGACCATCGATCCCATCCTGCGTGTGGTGACCCTGGCTAACACTGAAATCTCGCTCTCGACAGCTGATTTCGAATTTTGACAGCTAGCTCAGTCCTAGGTATAATACTAGTATCTGTTAGCCAGGGTGACTACGGTTTTAGAGCTAGAAATAGCAAGT	
csgD_qPCR_F	CCCGTACCGCGACATTG	ner for qPCR of cs
csgD_qPCR_R	ACGTTCTTGATCCTCCATGGA	

Table S3 Library plasmid construction primers

Name	Sequence
bbF	CCAGAAATCATCCTTAGCGAAAGCTAAGGATTTTTTTATCTGAAATTC
bbR	GTTTTAGAGCTAGAAATAGCAAGTTAAAATAAGGC
Insert_R	ACTTTTTCAAGTTGATAACGGACTAGCCTTATTTTAACTTGCTATTTCTAGCTCTAAAAC
BC1_F	agataaaaaaaatccttagctttcgctaaggatgatttctggTCCTCTGGCGGAAAGCCT
BC2_F	agataaaaaaaatccttagctttcgctaaggatgatttctggTTCGGATCGCAGGCTGCA
BC3_F	agataaaaaaaatccttagctttcgctaaggatgatttctggCCTGTCTTGGCGGACACA
BC4_F	agataaaaaaaatccttagctttcgctaaggatgatttctggAAGAGCACCGCCTTCGTC
BC5_F	agataaaaaaaatccttagctttcgctaaggatgatttctggTTCCAGCTCGAAGGCGAT
BC6_F	agataaaaaaaatccttagctttcgctaaggatgatttctggGCGGTGGCATTTTCAGGG
BC7_F	agataaaaaaaatccttagctttcgctaaggatgatttctggGAATCCTCCCGAGCCCAC
BC8_F	agataaaaaaaatccttagctttcgctaaggatgatttctggGCGCCTTCCTCGCTATGA
BC9_F	agataaaaaaaatccttagctttcgctaaggatgatttctggCGGGTTGCACGCCGAGTT
BC10_F	agataaaaaaaatccttagctttcgctaaggatgatttctggGGCCGGCAAAGACACTTG
BC11_F	agataaaaaaaatccttagctttcgctaaggatgatttctggGTTCGCCGGAGAATCCCT
BC12_F	agataaaaaaaatccttagctttcgctaaggatgatttctggTCGTTGTGAAGAACGCGC
BC13_F	agataaaaaaaatccttagctttcgctaaggatgatttctggTGAGGGCGAAGACAAGCA
BC14_F	agataaaaaaaatccttagctttcgctaaggatgatttctggCTTCTGTCGCCCTCTGGC
BC15_F	agataaaaaaaatccttagctttcgctaaggatgatttctggGGCCGGCAAAGACACTTG
BC16_F	agataaaaaaaatccttagctttcgctaaggatgatttctggGGCGGGCTCGGGCGGGAT
BC17_F	agataaaaaaaatccttagctttcgctaaggatgatttctggGTTCGCCGGAGAATCCCT
BC18_F	agataaaaaaaatccttagctttcgctaaggatgatttctggTTTCCTTCGGCGAAGGCA
BC19_F	agataaaaaaaatccttagctttcgctaaggatgatttctggTCGTTGTGAAGAACGCGC
BC20_F	agataaaaaaaatccttagctttcgctaaggatgatttctggCCCTGAGTCCTCCGCTGC
BC21_F	agataaaaaaaatccttagctttcgctaaggatgatttctggGTTCGGAAGGTGCCCTCA
BC22_F	agataaaaaaaatccttagctttcgctaaggatgatttctggGGGTGGCGGCTGTCTCTT
BC23_F	agataaaaaaaatccttagctttcgctaaggatgatttctggGCTGGAGGGTTGCCTGGG

Table S4 Library sequencing primers

Name	Sequence	Barcode
**Sequencing_primers**		
Index	CCGTTATCAACTTGAAAAAGTGGCACCGAGT	N/A
Read1	gttccttagctttcgctaaggatgatttctgg	N/A
Read2	ACTCGGTGCCACTTTTTCAAGTTGATAACGG	N/A
**Amplification primers**		
F1	AATGATACGGCGACCACCGAGATCTACACTATGGTAATTgttccttagctttcgctaaggatgatttctgg	N/A
BC1	CAAGCAGAAGACGGCATACGAGATacgagactgattAACTCGGTGCCACTTTTTCAAGTT	AATCAGTCTCGT
BC2	CAAGCAGAAGACGGCATACGAGATgctgtacggattAACTCGGTGCCACTTTTTCAAGTT	AATCCGTACAGC
BC3	CAAGCAGAAGACGGCATACGAGATatcaccaggtgtAACTCGGTGCCACTTTTTCAAGTT	ACACCTGGTGAT
BC4	CAAGCAGAAGACGGCATACGAGATtggtcaacgataAACTCGGTGCCACTTTTTCAAGTT	TATCGTTGACCA
BC5	CAAGCAGAAGACGGCATACGAGATatcgcacagtaaAACTCGGTGCCACTTTTTCAAGTT	TTACTGTGCGAT
BC6	CAAGCAGAAGACGGCATACGAGATgtcgtgtagcctAACTCGGTGCCACTTTTTCAAGTT	AGGCTACACGAC
BC7	CAAGCAGAAGACGGCATACGAGATagcggaggttagAACTCGGTGCCACTTTTTCAAGTT	CTAACCTCCGCT
BC8	CAAGCAGAAGACGGCATACGAGATaattgtgtcggaAACTCGGTGCCACTTTTTCAAGTT	TCCGACACAATT
BC9	CAAGCAGAAGACGGCATACGAGATtgcatacactggAACTCGGTGCCACTTTTTCAAGTT	CCAGTGTATGCA
BC10	CAAGCAGAAGACGGCATACGAGATagtcgaacgaggAACTCGGTGCCACTTTTTCAAGTT	CCTCGTTCGACT
BC11	CAAGCAGAAGACGGCATACGAGATaccagtgactcaAACTCGGTGCCACTTTTTCAAGTT	TGAGTCACTGGT
BC12	CAAGCAGAAGACGGCATACGAGATgtagatcgtgtaAACTCGGTGCCACTTTTTCAAGTT	TACACGATCTAC
BC13	CAAGCAGAAGACGGCATACGAGATtaacgtgtgtgcAACTCGGTGCCACTTTTTCAAGTT	GCACACACGTTA

BC14	CAAGCAGAAGACGGCATACGAGATcattatggcgtgAACTCG GTGCCACTTTTTCAAGTT	CACGCC ATAATG
BC15	CAAGCAGAAGACGGCATACGAGATccaatacgcctgAACTCG GTGCCACTTTTTCAAGTT	CAGGCG TATTGG
BC16	CAAGCAGAAGACGGCATACGAGATgatctgcgatccAACTCG GTGCCACTTTTTCAAGTT	GGATCG CAGATC
BC17	CAAGCAGAAGACGGCATACGAGATcaaacaacagctAACTC GGTGCCACTTTTTCAAGTT	AGCTGTT GTTTG
BC18	CAAGCAGAAGACGGCATACGAGATcgagcaatcctaAACTCG GTGCCACTTTTTCAAGTT	TAGGATT GCTCG
BC19	CAAGCAGAAGACGGCATACGAGATgcagaacgatatAACTC GGTGCCACTTTTTCAAGTT	ATATCGT TCTGC
BC20	CAAGCAGAAGACGGCATACGAGATtggaaggtgcacAACTC GGTGCCACTTTTTCAAGTT	GTGCACC TTCCA
BC21	CAAGCAGAAGACGGCATACGAGATtgtctgcctgtgAACTCG GTGCCACTTTTTCAAGTT	CACAGG CAGACA
BC22	CAAGCAGAAGACGGCATACGAGATcgtgtgtcctttAACTCG GTGCCACTTTTTCAAGTT	AAAGGA CACACG
BC23	CAAGCAGAAGACGGCATACGAGATttaccactcaagAACTCG GTGCCACTTTTTCAAGTT	CTTGAGT GGTAA
BC24	CAAGCAGAAGACGGCATACGAGATcttcacgttgtaAACTCG GTGCCACTTTTTCAAGTT	TACAAC GTGAAG
BC25	CAAGCAGAAGACGGCATACGAGATttgggtcgttggAACTCG GTGCCACTTTTTCAAGTT	CCAACG ACCCAA
BC26	CAAGCAGAAGACGGCATACGAGATaactatggcaacAACTCG GTGCCACTTTTTCAAGTT	GTTGCCA TAGTT
BC27	CAAGCAGAAGACGGCATACGAGATagacttcaatgcAACTCG GTGCCACTTTTTCAAGTT	GCATTGA AGTCT
BC28	CAAGCAGAAGACGGCATACGAGATgtctaggaccacAACTC GGTGCCACTTTTTCAAGTT	GTGGTCC TAGAC
BC29	CAAGCAGAAGACGGCATACGAGATtggaccactgttAACTCG GTGCCACTTTTTCAAGTT	AACAGT GGTCCA
BC30	CAAGCAGAAGACGGCATACGAGATtttagcgggtggAACTCG GTGCCACTTTTTCAAGTT	CCACCCG CTAAA
